# Fall Risk and Balance Confidence in Patients With Diabetic Peripheral Neuropathy: An Observational Study

**DOI:** 10.3389/fendo.2020.573804

**Published:** 2020-10-23

**Authors:** Tessa Riandini, Eric Y. H. Khoo, Bee Choo Tai, Subramaniam Tavintharan, Melissa S. L. A. Phua, Kurumbian Chandran, Siew Wai Hwang, Kavita Venkataraman

**Affiliations:** ^1^ Saw Swee Hock School of Public Health, National University of Singapore, Singapore, Singapore; ^2^ Department of Medicine, Yong Loo Lin School of Medicine, National University of Singapore, Singapore, Singapore; ^3^ Diabetes Centre, Khoo Teck Puat Hospital, Singapore, Singapore; ^4^ Podiatry Service, Tan Tock Seng Hospital, Singapore, Singapore; ^5^ Department of Medicine, Ng Teng Fong General Hospital, Singapore, Singapore; ^6^ SingHealth Polyclinics-Bukit Merah, Singapore, Singapore

**Keywords:** type 2 diabetes, diabetic**** neuropathy, falls, fall risk, balance confidence

## Abstract

**Objective:**

Individuals with diabetic peripheral neuropathy (DPN) have functional deficits that increase their risk of falling. However, psychological aspects such as loss of confidence in undertaking activities could also contribute to this risk. We examined correlations between balance confidence and fall risk among individuals with DPN.

**Methods:**

This was a cross-sectional study of 146 individuals with DPN. Elevated fall risk was determined by timed up-and-go test with standard cut-off time of 13.5 seconds, and balance confidence was measured by 16-item Activities Specific Balance Confidence scale. Functional parameters assessed included functional reach, body sway velocity during quiet standing and muscle strength at ankle and toe.

**Results:**

Twenty percent of the DPN patients were at increased risk of falls. Every unit increase in balance confidence was associated with 9% (95% confidence interval: 0.88, 0.95; p<0.001) reduced odds of falling, after adjusting for socio-demographic, health and functional characteristics. No other functional parameters had significant associations with fall risk in adjusted analyses.

**Conclusions:**

Psychological factors like balance confidence appear to be more important for fall risk among DPN patients, compared to objective functional performance. Interventions targeting balance confidence may be beneficial in reducing the risk of falls in this population.

## Introduction

Diabetic peripheral neuropathy (DPN) may lead to muscle weakness, loss of ankle reflexes, and impairment in balance, coordination and gait control (1), which significantly increase the risk of falling and sustaining fall-related injuries (2–4). On the other hand, patients with DPN, regardless of their DPN severity, have been shown to have an increased fear of falling (5), which may lead to avoidance of tasks within their capabilities and therefore decline in mobility and increased risk of falling. It has been hypothesized, therefore, that both sensorimotor function and cognitive processes such as fear of falling contribute to fall risk (6). Fear of falling could originate from individuals’ own perception and confidence of their ability to maintain balance (7). Low confidence in undertaking activities is prevalent among diabetic patients, (5) and could be more common in DPN patients given their higher rate of alterations in balance and gait. However, it is not clear if lower balance confidence is associated with higher fall risk among DPN patients. In this study, we examined relationships between balance confidence, balance performance and fall risk among DPN patients.

## Materials and Methods

### Design and Participants

This study was approved by the National Healthcare Group Domain Specific Review Board and SingHealth Centralized Institutional Review Board. A total of 236 patients with physician-diagnosed type 2 diabetes mellitus were recruited from outpatient clinics at 5 centers across Singapore between July 2014 and April 2017. Individuals with active foot problems and unrelated orthopedic, surgical, or medical conditions affecting functional mobility and balance, including poor visual acuity and severe diabetic retinopathy, were excluded. More details of the study methodology have been published elsewhere (8). All eligible patients provided written informed consent prior to participation.

### Outcome Measures

The present study was restricted to 146 patients who had DPN, defined by neurothesiometer reading of >25 V, and/or positive monofilament test in two or more sites in either foot, and/or presence of DPN symptoms as measured by Michigan Neuropathy Screening Instrument (9). Fall risk was evaluated using the timed up-and-go (TUG) test (10). A cut-off of 13.5 seconds on the TUG test was used to define high fall risk (11).

### Functional Assessment

We used 16-item Activities Specific Balance Confidence (ABC) scale to assess participants’ confidence in performing daily or routine activities without losing their balance (12). Static balance was assessed by measuring body sway velocity on quiet standing with eyes closed for two minutes using a balance platform (Accugait AMTI, USA). Dynamic balance was assessed using the functional reach test (13). Muscle strength was measured at the ankle and great toe using a handheld dynamometer. Hours of walking per week was derived from the International Physical Activity Questionnaire.

### Other Covariates

Two classes of potential confounders were evaluated. Socio-demographic characteristics included age, gender, ethnicity, marital, education, employment status, and housing size. Health-related measures were body mass index (BMI), HbA1c levels, diabetes duration, DPN medication, and self-reported comorbid conditions (high blood pressure, high cholesterol, heart disease, and arthritis). Health utility index was derived from self-administered EQ-5D-5L questionnaire, with scores converted into a single index using the value set for Japan (14).

### Data Analysis

All socio-demographic, health and functional parameters between DPN patients at high and low fall risk were compared using two-sample t tests for continuous variables and chi-square tests for categorical variables. Bivariate associations with fall risk were examined using logistic regression models for each parameter independently (crude models). Forward and backward stepwise logistic regression models were then constructed to identify the final multivariable model with the best fit by Akaike’s Information Criteria (AIC). Goodness-of-fit of the final model was assessed by Hosmer-Lemeshow test. All analyses were performed in R Software, version 3.5.1 (R Development Core Team, Vienna).

## Results

On average, our DPN patients were in their early 60s, and majority were Indians (**Table 1**). One in five DPN patients were at increased fall risk. On bivariate analysis, 1-unit increase in balance confidence was significantly associated with a 7% (OR=0.93, 95% CI: 0.91, 0.95) reduced odds of falling among DPN patients. Time spent on walking, health utility score, functional reach, great toe extensor strength, diabetes duration, BMI, gender, education, working status and housing status were also associated with fall risk on bivariate analysis.

**Table 1:** Characteristics of participants (n=146).

Characteristics^†^	High risk (n = 29)	Low risk (n = 117)	p-value^‡^
***Socio-demographic status***			
Age [mean (SD), in years]	63.98 (6.36)	61.68 (6.91)	0.106
Female	22 (75.9)	60 (51.3)	**0.029**
Indian ethnicity	24 (82.8)	87 (74.4)	0.480
Married	16 (55.2)	81 (69.2)	0.224
Secondary education or above	13 (44.8)	80 (68.4)	**0.032**
Currently employed	6 (20.7)	61 (52.1)	**0.005**
Living in 4-room apartment or bigger	10 (35.7)	68 (58.6)	**0.049**
***Health measures***			
BMI [mean (SD), in kg/m^2^)	31.32 (7.11)	27.73 (5.26)	**0.003**
HbA1c [mean (SD), in %]	8.87 (1.99)	8.42 (1.80)	0.237
Diabetes duration [mean (SD), in years]	21.90 (10.97)	13.95 (10.34)	**<0.001**
High blood pressure	26 (89.7)	77 (65.8)	**0.022**
High cholesterol	23 (79.3)	77 (65.8)	0.239
Heart disease	9 (31.0)	26 (22.2)	0.452
Arthritis	6 (20.7)	11 (9.4)	0.170
Health utility score	0.60 (0.13)	0.75 (0.16)	**<0.001**
On DPN medication	1 (3.4)	4 (3.4)	1.000
Experienced fall in the past 4 weeks	1 (3.4)	3 (2.6)	1.000
***Functional measures*** *[mean (SD)]*			
Walking per week (h)	19.45 (14.62)	26.61 (16.42)	**0.033**
Total ABC	48.85 (18.98)	80.95 (17.49)	**<0.001**
Functional reach (cm)	19.38 (6.24)	25.23 (6.63)	**<0.001**
Body sway velocity (mm/s)	1.95 (1.28)	1.61 (1.17)	0.169
Ankle dorsiflexion strength (lb)	9.98 (2.69)	11.18 (3.17)	0.062
Great toe extensor strength (lb)	5.90 (1.48)	7.03 (1.79)	**0.002**

ABC, activities^-^based balance confidence; BMI, body mass index; DPN, diabetic peripheral neuropathy; SD, standard deviation.

^†^Unless otherwise specified, statistics presented are n (%).

^‡^Bold text indicates statistically significant difference with p < 0.05.

Balance confidence, working status, BMI, diabetes duration, health utility score, functional reach and great toe extensor strength, were included in the final multivariable model (minimum AIC: 79.5; Hosmer-Lemeshow p=0.36). Balance confidence remained significantly associated with fall risk, with 9% (95% CI: 0.88, 0.95) lower odds of falling per unit increment of total ABC score, after adjusting for other confounders (**Table 2**). Diabetes duration (OR=1.14, 95% CI: 1.05, 1.23) was also associated with fall risk after adjusting for other parameters, though the effect estimates of working status, BMI, health utility score, functional reach and great toe extensor strength became non-significant in the final multivariable model.

**Table 2:** Factors associated with increased fall risk among DPN patients (n=146).

	Model 1^†^			Model 2^‡^		
	OR	95% CI	p-value^§^	OR	95% CI	p-value^§^
***Socio-demographic status***						
Age	1.05	(0.99, 1.12)	0.108	–		–
Gender, female	**2.99**	**(1.10, 7.53)**	**0.020**	–		–
Ethnicity, Indian	1.66	(0.58, 4.73)	0.346	–		–
Marital status, not married	1.83	(0.80, 4.19)	0.155	–		–
Education, primary or below	**2.66**	**(1.16, 6.10)**	**0.021**	–		–
Working status, unemployed	**4.18**	**(1.58, 11.0)**	**0.004**	2.75	(0.68, 11.14)	0.156
Housing, 3-room apartment or smaller	**2.35**	**(1.02, 5.43)**	**0.045**	–		–
***Health measures***						
BMI (kg/m^2^)	**1.11**	**(1.03, 1.19)**	**0.004**	1.12	(0.99, 1.26)	0.071
HbA1c (%)	1.14	(0.92, 1.41)	0.237	–		–
Diabetes duration (years)	**1.07**	**(1.03, 1.11)**	**0.001**	**1.14**	**(1.05, 1.23)**	**0.001**
High blood pressure	**4.50**	**(1.28, 15.79)**	**0.019**	–		–
High cholesterol	1.99	(0.75, 5.29)	0.167	–		–
Heart disease	1.58	(0.64, 3.87)	0.322	–		–
Arthritis	2.51	(0.84, 7.49)	0.098	–		–
Walking per week (min)	**0.97**	**(0.94, 1.00)**	**0.036**	–		–
***Functional measures***						
Health utility score (per 0.01 unit)	**0.92**	**(0.89, 0.96)**	**<0.001**	0.96	(0.91, 1.01)	0.109
Total ABC	**0.93**	**(0.91, 0.95)**	**<0.001**	**0.91**	**(0.88, 0.95)**	**<0.001**
Functional reach	**0.87**	**(0.81, 0.93)**	**<0.001**	0.92	(0.83, 1.01)	0.085
Body sway velocity	1.24	(0.91, 1.69)	0.175	–		–
Ankle dorsiflexion strength	0.88	(0.76, 1.01)	0.065	–		–
Great toe extensor strength	**0.65**	**(0.49, 0.86)**	**0.003**	0.67	(0.42, 1.07)	0.093

ABC, activities-based balance confidence; BMI, body mass index; DPN, diabetic peripheral neuropathy; SD, standard deviation.

^†^Model 1: Crude models.

^‡^Model 2: Adjusted model, stepwise forward and backward selections. Variables considered: gender, education, working status, housing status, BMI, diabetes duration, high blood pressure, walking per week, health utility score, total ABC, functional reach and great toe extensor strength (Hosmer-Lemeshow test p-value: 0.362).

^§^Bold text indicates a statistically significant difference with a p < 0.05.

## Discussion

To our knowledge, this is the first study to examine association between balance confidence and fall risk among DPN patients. We observed that improvement in balance confidence, measured by total ABC, was associated with a reduction in fall risk, irrespective of other functional and health measures. In fact, objectively assessed measures of balance performance, i.e. body sway velocity and functional reach, were not significantly associated with fall risk in our final model. This finding is consistent with the hypothesis that lower balance confidence and fear of falling significantly restrict one’s daily activities (15), which could contribute to physical deconditioning, and lead to increased levels of disability and loss of independence that could elevate fall risk (16, 17). Our finding is also in line with existing literature that reported inverse relationship between balance confidence and fall risk in older adults (17, 18), and further supports the growing evidence linking diabetes and DPN to balance impairment, and consequently increase in fall risks (8, 19). While exercise/physical therapy interventions have been shown to improve physical functioning in people with DPN (20), only a few of these interventions have been reported to also result in improved balance confidence concurrently (21). We are not aware of any studies in people with DPN that have targeted improvement in balance confidence in order to reduce fall risk, though longitudinal studies from other populations indicate that poor balance confidence predicts future levels of physical functioning and disability (22, 23). Therefore, balance confidence could be an important target for intervention in people with DPN as well for maintenance of physical activity levels and reducing fall risk in the long-term, and this should be formally examined in future studies.

We also observed positive associations between diabetes duration and fall risk. Prolonged duration of diabetes has previously been shown to be associated with muscle weakness (24), and increased risk of fall-related hospitalization (25).

Some study limitations should be considered. Our study may overestimate fall risk in DPN patients as the definition of DPN used predominantly identifies those with advanced neuropathy at the risk of ulceration, and may therefore exclude those with early or small fibre neuropathy. DPN was considered as a binary variable (yes or no), therefore we are unable to comment on any association between DPN severity and fall risk. Indians and women are also disproportionately represented in our study, which may compromise the external but not internal validity of our observations. There is potential for selection bias due to the voluntary nature of the study; nevertheless, only 10 of the 166 participants with DPN screened declined participation, and the rest were ineligible. While TUG is a recommended routine screening test for falls (26), its predictive accuracy in identifying fall risk is limited, which may be explained by the fact that TUG only captures strength, balance and mobility, but does not encompass multiple intrinsic and extrinsic factors (e.g., lifestyle, medication, environment) that the risk of falling depends on (27, 28). Data on other medications, and on mental well-being (apart from the extent captured by the EQ5D) were not collected in this study. However, we used a comprehensive set of objective and self-reported measurements of health and functional status, which is a strength. Adjustment for these measures did not eliminate the significant association observed between balance confidence and fall risk.

In summary, increased balance confidence is associated with reduction in fall risk among DPN patients, which suggests the importance of psychological factors in falls prevention. This highlights the potential role of interventions targeting improvements in balance confidence to reduce fall risk. More research is needed to understand how balance confidence affects fall risk and identify effective interventions.

## Data Availability Statement

The raw data supporting the conclusions of this article will be made available by the authors, without undue reservation.

## Ethics Statement

The studies involving human participants were reviewed and approved by National Healthcare Group Domain Specific Review Board and SingHealth Centralized Institutional Review Board. The patients/participants provided their written informed consent to participate in this study.

## Author Contributions

EK, BT, ST, MP, KC, SH, and KV contributed to the research idea and design. TR, EK, BT, ST, MP, KC, SH, and KV contributed to data collection. TR and KV contributed to data analysis and interpretation. TR drafted the manuscript. KV revised the manuscript and prepared the final version for submission. All authors contributed to the article and approved the submitted version.

## Funding

This work was supported by the National Medical Research Council, Singapore (grant number NMRC/TA/0022/2014).

## Conflict of Interest

The authors declare that the research was conducted in the absence of any commercial or financial relationships that could be construed as a potential conflict of interest.
